# Chemoprevention of elite tea variety CFT‐1 rich in EGCG against chemically induced liver cancer in rats

**DOI:** 10.1002/fsn3.1121

**Published:** 2019-07-04

**Authors:** Sufeng Liao, Jinke Lin, Jianghong Liu, Tuansheng Chen, Ming Xu, Jingui Zheng

**Affiliations:** ^1^ Agricultural Product Quality Institute Fujian Agriculture and Forestry University Fuzhou China; ^2^ Key Laboratory of Ministry of Education for Genetics, Breeding and Multiple Utilization of Crops, College of Crop Science Fujian Agriculture and Forestry University Fuzhou China; ^3^ Anxi College of Tea Science Fujian Agriculture and Forestry University Fuzhou China; ^4^ Hospital of Fujian Agriculture and Forestry University Fuzhou China; ^5^ Key Laboratory of Fujian Province for Crop Biotechnology Fujian Agriculture and Forestry University Fuzhou China

**Keywords:** *Camellia sinensis* (L.) O. Kuntze cv. CFT‐1, chemoprevention, EGCG‐rich tea variety, liver cancer, *N*‐nitrosodiethylamine

## Abstract

*Camellia sinensis* (L.) O. Kuntze cv. CFT‐1 is an elite tea variety bred by sexual hybridization with a high content of epigallocatechin‐3‐gallate (EGCG) as 134.2 mg/g (which is 2.54‐fold that of the common variety). This study was to evaluate the chemopreventive effects of CFT‐1 green tea infusion (CFT‐1) against *N*‐nitrosodiethylamine (NDEA)‐induced hepatocarcinogenesis in rats and its mechanisms. The results showed that CFT‐1 had a superior inhibitory effect in NDEA‐initiated hepatocarcinogenesis compared to that of common tea. CFT‐1 significantly reduced the hepatic nodules incidence, size, and number and prevented the hepatic adenoma or hepatocellular carcinoma (HCC) formation. In particular, CFT‐1‐treated animals had the least incidence of HCC (8.33%) followed by common tea treatment (40.00%) and model control rats (87.50%). CFT‐1 treatment significantly ameliorated abnormal liver function enzymes, reduced serum AFP, CEA, TSGF, and TNF‐α levels, inhibited the dickkopf‐related protein‐1 expression, enhanced antioxidant capacity, suppressed the production of livers 8‐hydroxy‐2′‐deoxyguanosine, and regulated hepatic phase I and II xenobiotic‐metabolizing enzymes. Transcriptomic analysis of liver tissue suggested that compared to common tea, administration of CFT‐1 regulated larger gene sets, which were located in several important pathways of antioxidants, inflammatory network, xenobiotic‐metabolizing enzymes, apoptosis, cell proliferation, and metabolism associated with liver tumorigenesis. We identified some genes as potential molecular targets involved in the prevention of CFT‐1 and found that CFT‐1 inhibited inflammation response, proliferation, and accelerated apoptosis by inhibiting NF‐κB and PI3K/Akt pathway. Thus, EGCG‐rich CFT‐1 green tea might be a potential choice for liver cancer prevention/treatment in the future.

## 1 INTRODUCTION

Primary liver cancer is one of the most frequent malignant tumors, representing the fifth most common malignancy worldwide and the second leading cause of cancer deaths, with a 5‐year survival rate of 17% (Fujise et al., 2016; Llovet et al., 2012). China has more than 55% liver cancer cases globally. Recent studies have shown that the side effects of chemical drugs for HCC treatment is still not be neglected (Shen et al., 2010). Hence, better chemopreventive agents for HCC with fewer side effects are desired.

Tea made from the fresh leaf of *Camellia sinensis*. The anticancer effects of green tea and its products are supported by a large amount of evidence for cell culture and animal models research (Fujikia & Suganuma, 2012; Kou, Kirberger, Yang, & Chen, 2013). Furthermore, epidemiological studies indicated that dietary intake of tea may prevent and reduce the risk of different types of malignancies, including liver cancer (Rady, Mohamed, Rady, Siddiqui, & Mukhtar, 2018). These beneficial effects of green tea are primarily attributed to the presence of a type of polyphenols known as catechins and its monomer, including epigallocatechin‐3‐gallate (EGCG), epigallocatechin (EGC), epicatechin gallate (ECG), epicatechin (EC), and catechin. Hence, the chemical composition and content of phytochemicals in tea are crucial to its biological activity.

As the predominant component (25%–55% catechins content) of tea (Du et al., 2012; Lin, Zheng, Chen, & Chen, 2005), the concentration of EGCG in tea or tea products on the market was generally no more than 50.0 mg/g (Zheng, Huang, Huang, & Chen, 2007). Thus, we screened and bred some elite tea varieties, with high contents of EGCG (>100 mg/g) from 3,357 tea plant germplasm resources, such as *C. sinensis* (L.) O. Kuntze “1005,” “E‐101,” “033,” “505,” “509,” “524,” and “09‐5” in our previous work (Lin et al., 2005; Zheng et al., 2012). From these progenies of EGCG‐rich tea varieties, we had bred a new tea variety by sexual hybridization, *Camellia sinensis* (L.) O. Kuntze cv. CFT‐1, which was rich in EGCG (134.2 mg/g, 2.54‐fold that of the common variety *Fuyun6* green tea), as well as rich in total catechins, ECG, EGC, and catechin (Liao, Liu, Yang, & Zheng, 2017). Our previous study have found that the CFT‐1 green tea extract has better hypolipidemic and hepatoprotective effects compared to the common tea (Liao et al., 2017), as well as more effective anticancer effect in HepG2 cell, SK‐OV‐3 cell, Hela cell, A549 cell, U‐87MG cell, BT‐474 cell, PC‐3 cell, HL‐60 cell, SMMC‐7721 cell, HCT‐116 cell, A431 cell, K562 cell, PANC‐1 cell, and BGC‐823 cell than EGCG alone or common tea (Zheng et al., 2016). However, whether the CFT‐1 has more efficiency on cancer prevention than that of common tea remains unknown.

Some mechanisms for modulating cancer signaling and metabolic pathways with EGCG have been proposed based on numerous studies in cells, such as TNF, PI3K/Akt/mTOR, p53, p38 MAPK, and NF‐κB pathways (Chan, Lee, Wang, Yeh, & Liang, 2018; Elguindy, Yacout, El Azab, & Maghraby, 2016; Li et al., 2016; Rady et al., 2018; Wang, Wang, Wan, Yang, & Zhang, 2015). Our previous studies also found that EGCG, as well as EC, ECG, and EGC, may bind to some cancer‐related proteins in TPK‐RAS‐MAPK and NF‐κB signaling pathways when used molecular docking of these bioactive substances (Zheng, Chen, & Lu, 2011). Nevertheless, it remains unclear whether these mechanisms contributed to the cancer preventive effects in vivo.

Therefore, in this study, we evaluated the chemopreventive properties of EGCG‐rich tea CFT‐1 on *N*‐nitrosodiethylamine (NDEA)‐induced HCC and its underlying mechanisms.

## 2 MATERIALS AND METHODS

### 2.1 Chemicals

*N*‐nitrosodiethylamine, EGCG, EGC, ECG, EC, C, gallocatechin (GC), catechin gallate (CG), gallocatechin gallate (GCG), and caffeine (at least 98% purity) were purchased from Sigma‐Aldrich. The immunohistochemical SP‐HRP kit, ECL plus Western blot kit purchased from Dingguo Biological Technology. All other chemicals were purchased from local commercial sources in China.

### 2.2 Preparation of EGCG‐rich green tea

Tea plants of *Camellia sinensis* (L.) O. Kuntze cv. CFT‐1 was cultivated by our institution in 2012 at Fujian, Fujian Province. The common tea *Camellia sinensis* (L.) O. Kuntze cv. *Fuyun6* (an elite tea variety approved by National Crop Variety Approval Committee in China in 1987, No.: GS13033‐1987) was introduced from the original planting area and currently preserved as a tea germplasms resource in our institute. To do animal experiments, “one and two bud” young shoots were harvested from tea plants in June 2015. Samples were fixed with hot air at 105°C for 6 min and dried at 90°C until water content is lower than 5%, then made into green tea and stored at −20°C.

### 2.3 Preparation of tea infusion and composition analysis

CFT‐1 green tea infusion (CFT‐1) and *Fuyun6* green tea infusion (FYT) were prepared as follows: briefly, 30 g tea powders were extracted with 1 L distilled water (80°C) for 6 min, then ultrasonic extraction for 15 min with power of 200 W. Subsequently, the extraction was filtered by four layers of gauze and constant volume to 1 L of water. The resultant tea infusion (3:100, w/v) was collected and stored at −20°C.

Qualitation of total catechins, catechin monomers, and caffeine in tea infusions was performed by a LC–MS/MS (Thermo Fisher Scientific) with a MRM mode. The LC–MS analysis was carried out using a LC–MS system comprising of a LC (2010, Finnigan) and LCQ Fleet Ion Trap LC/MS (LCQ Fleet, Thermo Fisher Scientific). In the negative ion detection mode, using a peristaltic pump direct injection analysis ESI source mass spectrometry, the peristaltic pump flow rate: 5 µl/min, capillary temperature: 300°C, sheath gas (N_2_):15 arb, spray voltage: −4.47 kV, Ion detection voltage: 1.5 kV, Gauge pressure: 0.83 × 10^−5^ Torr, time: 100 ms, collision gas: He, width: 3 u, select the parent ion collection range: 100–400 m/z.

The chromatographic separation and quantification of the test phytochemicals in tea infusion was achieved using a ZORBAX Eclipse XDB‐Phenyl (4.6 mm × 250 mm, 5 μm) analytical column (Agilent Technologies, Santa Clara, CA, USA) on a Finnigan HPLC system (Thermo Fisher Scientific, Waltham, MA, USA) equipped with an auto‐sampler unit. The column temperature was 35°C. The mobile phase was delivered at a flow rate of 1 ml/min, consisting of 2% formic acid–water and 80% acetonitrile. The injection volume was 10 μl. The contents of total catechins, catechin monomers, and caffeine in samples were calculated with a standard calibration curve obtained from each authentic standard. Total polyphenol content was quantified using the Folin–Ciocalteu method (Chen et al., 2012). Tea polysaccharides were quantified using an anthrone‐sulfuric acid method (Cheng, Chen, Yang, Wang, & Wei, 2018). Theanine was measured according to Song's methods (2015).

### 2.4 Animals and experimental design

The study was conducted in strict accordance with the Chinese national guidelines for the care of laboratory animals and was approved by the Animal Ethics Committee of the Institute of Laboratory Animal Sciences at the Chinese Academy of Medical Sciences. Male Wistar rats (*N* = 48, 140–160 g) were obtained from Beijing WeiTong Lihua Experimental Animal Technology Co., Ltd. (animal production license No.: SCXK 2012‐0001, animal certificate number: 11400700135387). Rats were housed (4 per 43 × 27 × 15 cm cage) in a specific pathogen‐free animal room and maintained under standard experimental conditions, temperature 22 ± 1°C, and relative humidity 50 ± 10% with a 12 hr light/dark cycle. After 1 week quarantine, the rats were randomized into four groups (*N* = 12/group): normal control group (control group, i.p. of 0.9% saline solution twice a week from 2–9 weeks and drink water), model controls group (NDEA group, i.p. of 25 mg/kg NDEA twice a week from 2–9 weeks and drink water), CFT‐1‐treated group (NDEA + CFT‐1 group, i.p. of 25 mg/kg NDEA twice a week from 2–9 weeks and drink CFT‐1 for 20 weeks), FYT‐treated group (NDEA + FYT group, i.p. of 25 mg/kg NDEA twice a week from 2–9 weeks and drink FYT for 20 weeks). The feed, water, tea infusion intake, and body weights were measured weekly. After 20 weeks of the experiment, rats were anesthetized with i.p. pentobarbital sodium (35 mg/kg) and blood samples were collected for hematological and plasma biochemical analyses. After blood samples collection, rats were immediately sacrificed. Then, organs were excised, washed with ice‐cold saline, and weighed. The hepatic neoplasm‐related indicators were measured as described earlier (Chen, Myracle, Wallig, & Jeffery, 2016). Three liver samples from each group were collected and stored at −80°C for RNA sequencing.

### 2.5 Hematological and biochemical analysis

BC‐2800Vet Automatic Hematology Analyzer (Mindray Bio‐Medical Electronics Co., Ltd) was used to detect leukocytes, granulocytes, erythrocytes (RBC), and platelets. Plasma was immediately separated by centrifugation, and biochemical data for serum alanine transferase enzyme (ALT), gamma‐glutamyl transpeptidase (GGT), total bilirubin (TBil), lactate dehydrogenase (LDH), alkaline phosphatase (ALP), and aspartate aminotransferase (AST) were quantified using commercial detection kits (Nanjing Jiancheng Bioengineering Institute) according to the manufacturer's instructions by GENios (TECAN, Infinite M200 PRO). The serum total bile acide (TBA), alpha‐fetoprotein (AFP), carcino embryonie antigen (CEA), tumor specific growth factor (TSGF), and tumor necrosis factor α (TNF‐α) were measured using ELISA assay kits purchased from Cusabio Biotech Co., Ltd.

### 2.6 Histopathological analysis and hepatic ultrastructure analysis by electron microscopy

Hepatic tissues from each group were collected rapidly and were immediately fixed in 10% buffered formalin for 48 hr, followed by 70% ethanol and embedded in paraffin. Sections were stained with hematoxylin and eosin (H&E) according to Wang's methods (2016) and examined under a light microscope (Nikon E200, Nikon Corp.).

Hepatic samples were cut into cubes (3 mm × 3 mm × 3 mm) and fixed in 0.25 mol/L glutaraldehyde buffer at pH 6.5. The fixed samples were cut into 1–3 mm^3^ and were washed with 0.1 mol/L sodium carbonate buffer (pH 5.6) for three times and then postfixed in 0.1 mol/L osmium tetroxide for 2 hr at 4°C. Then, samples were washed in sodium cacodylate buffer (pH 7.4), dehydrated through a graded series of acetone, and were embedded in epoxy resin (EPON 812). The ultrathin sections (90 nm) were cut from the resin blocks with an ultramicrotome (MT‐500, DuPont), stained with 2% uranyl acetate followed by Sato's lead staining solution. Stained sections were then viewed using JEOL 1400 TEM (JEOL).

### 2.7 Antioxidant biomarkers and phase I, phase II enzymes analysis

Livers were homogenized by a Teflon pestle connected to a Braun homogenizer motor in the 50 mmol/L phosphate buffer (pH 7.0) of 0.1 mmol/L EDTA at 4°C, followed by centrifuged for 10 min at 4,000 *g*. The centrifugal supernatant was the 10% (w/v) tissue homogenate. The thiobarbituric acid‐reactive substances (TBARS) and 8‐hydroxy‐2′‐deoxyguanosine (8‐OH‐dG) concentrations of livers were measured with TBARS and 8‐OH‐dG ELISA Kit (Lifespan Biosciences, Inc.). Malondialdehyde (MDA) content in livers and activity of superoxide dismutase (SOD), catalase, and glutathione peroxidase (GSH‐Px) in serum and livers were quantified with assay kits (Nanjing Jiancheng Bioengineering Institute).

Microsomal fractions were prepared from livers as Zheng's methods (2018). The contents of cytochrome b5 (Cyt b5) and cytochrome P450 (Cyt P450) were measured as previously described (Zheng et al., 2018). The hepatic GST and UDP‐glucuronosyltransferase (UGT) concentrations were measured using ELISA kits (Jiang Lai Biotechnology Co., Ltd).

### 2.8 Immunohistochemistry analysis and TUNEL assays

Paraffin‐embedded tissue was sectioned (4 μm) and processed as described earlier (Kheradmand, Dezfoulian, & Alirezaei, 2012). The sections were incubated with the primary antibodies of dickkopf‐related protein‐1 (Dkk1) (ab93017, 1:1,000, Abcam Shanghai Trading Co., Ltd), CYP1E2 (1:400, Cell Signaling), 8‐OH‐dG (1:800, Becton, Dickinson and Company, Inc.), ptgs2 (1:1,000, Santa Cruz Biotechnology), NF‐κB (1:4,000, Cell Signaling Technology, Inc.), TNF‐α (1:1,000, Cell Signaling Technology, Inc.), and Bcl‐2 (1:500, Santa Cruz Biotechnology), PCNA (1:1,600, Cell Signaling Technology, Inc.) with 3% BSA‐PBS overnight at 4°C in a humidified chamber. Immunohistochemistry and image analysis were performed according to Arivazhagan and Sorimuthu's methods (2015), and a ratio of positive to negative cells was quantified. TUNEL‐positive cells were counted and normalized to liver cell number.

### 2.9 Western blotting analysis

Proteins from hepatic tissues were extracted using a protein extraction kit (CWBIO) and quantified using a BCA Protein assay kit (CWBIO) and subjected to Western blotting as follows: proteins after heat treatment were electrophoresed on 12% polyacrylamide gels electrophoresis and transferred onto polyvinylidene fluoride membrane (Millipore Corporation). Blots were blocked with 5% nonfat milk in buffer‐containing TBST (10 mM Tris, pH 7.4, 150 mM NaCl, and 0.1% Tween‐20) at RT for 1 hr and then incubated overnight at 4°C with specific antibodies, including p‐IKK‐α, p‐IκB‐α, NF‐κB, PCNA, TNF‐α, ptgs2, nod2, p‐PI3K, PI3K (P85), p‐Akt (S473), Akt, Bax, p53, Bcl‐2, caspase 3, and β‐actin in TBST with 5% nonfat milk. After washing with TBST three times, membranes were incubated with appropriate secondary antibodies (1:5,000) for 1.5 hr at RT; after extensive washing with TBST, the membranes were processed for chemiluminescence detection using ChemiDocXRS (Bio‐Rad Company) and an ECL Western blotting substrate kit.

### 2.10 RNA extraction

Total RNA was extracted from livers using the method of Zheng et al. (2018). RNA concentration was measured using Qubit^®^ RNA Assay Kit in Qubit^®^ 2.0 Flurometer (Life Technologies). RNA purity was checked using the NanoPhotometer^®^ spectrophotometer (NanoDrop 2000, Thermo Fisher Scientific). RNA degradation and contamination was monitored using 1% agarose gel electrophoresis. RNA integrity was assessed using the RNA Nano 6000 Assay Kit of the Agilent 2100 Bioanalyzer (Agilent Technologies). A total amount of 3 μg RNA per sample was used as input material for the RNA sample preparations to construct RNA‐seq libraries. Three biological replicates were prepared in each sample.

### 2.11 cDNA library preparation for transcriptome sequencing

The clustering and sequencing of RNA samples were performed by the Novogene Experimental Department of Novogene Ltd. After cluster generation, the sequencing libraries were sequenced using NEBNext^®^ Ultra™ RNA Library Prep Kit on Illumina Hiseq X‐ten platform (Illumina, Inc.), and 150‐bp paired‐end reads were generated. According to a report published by Bolger, Lohse, and Usadel (2014), illumine paired‐end reads from each sample were first processed to remove adaptor and low‐quality reads with Trimmomatic. High‐quality reads were then mapped to reference genome with Hisat2 (Pertea, Kim, Pertea, Leek, & Salzberg, 2016). Mapped reads were filtered to remove PCR duplicates, uniquely mapped reads were used to count the reads in reference genome, and expression of genes was normalized to Fragments Per Kilobase of transcript per Million fragments mapped (FPKM) (Anders, Pyl, & Huber, 2015). Raw data are publicly available through GEO accession number GSE126796. Differential expression analysis was identified using the DEGSeq R package (Wang, Feng, Wang, Wang, & Zhang, 2009) between control group and each treated group by a strict algorithm modified from a Poisson distribution method. The differentially expressed genes (DEGs) were determined according to ranking by fold change of expression values in reads per million after filtering genes with *p*‐values. The resulting *p*‐values were adjusted using the Benjamini and Hochberg's method for controlling the false discovery rate. Genes with a corrected *p*‐value <0.05 were assigned as statistically significant.

### 2.12 GO and KEGG enrichment analysis of DEGs

Differentially expressed genes were then subjected to the “Core analysis” function by Ingenuity Pathway Analysis (IPA) software (IPA 4.0, Ingenuity Systems, www.Ingenuity.com). GO enrichment analysis of DEGs was implemented by TopGO package (Alexa & Rahnenfuhrer, 2010). GO terms with *p*‐value <0.05 were identified as significant. DEGs were also evaluated in KEGG database for enrichment with KOBAS software (Xie et al., 2011). All pathways with *p*‐value <0.05 were presented in Appendix S2.

### 2.13 qRT‐PCR

Total RNA was prepared according to chapter 2.10. cDNA was synthesized from 500 ng of RNA using HiScript Q RT Super Mix (TaKaRa). cDNA was subjected to real‐time qPCR amplification with AceQTM qPCR SYBR Green Master Mix (TaKaRa) using an ABI 7500 Sequence Detection System (Applied Biosystems Co., Ltd.). Each sample was analyzed in triplicate in three independent experiments. Sequences of the primers are listed in Table S1 of Appendix S1. The mRNA expression was defined from the threshold cycle, and relative expression was calculated using the 2^−ΔΔCT^ method after normalization to the internal reference GAPDH expression (Sur et al., 2016).

### 2.14 Statistical analysis

All data were expressed as mean ± *SD*. Statistical significance was assessed with ANOVA, Student's *t *test, χ^2^ test, chi‐squared test, Fisher's exact, or Wilcoxon test (SPSS 19.0 software). *p* < 0.05 was considered as statistically significant.

## 3 RESULTS

### 3.1 Phytochemical analysis

**Figure 1:**
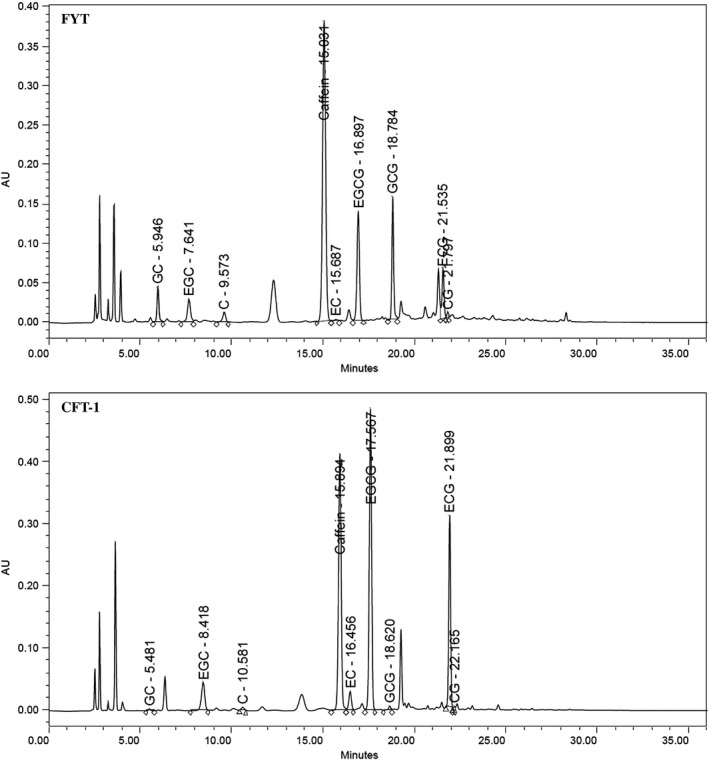
The HPLC chromatograms of CFT‐1 and Fuyun6 green tea infusion. Night major peaks were detected. C, catechin; EC, epicatechin; ECG, (−)‐epicatechin‐3‐gallate; EGC, (−)‐epigallocatechin; EGCG, (−)‐epigallocatechin‐3‐gallate; GCG, (−)‐gallocatechin‐3‐gallate

The typical HPLC chromatograms of CFT‐1 and FYT green tea infusion (3:100, w/v) are exhibited in Figure 1, while characterization of chemical constituents of CFT‐1 green tea infusion by MS is shown in Table S2 of Appendix S1. The contents of eight catechin monomers, caffeine, total catechin, and polyphenols in CFT‐1 green tea infusion were significantly higher than that of common green tea FYT (Table 1), and these distinct differences are consistent with our previous study (Liao et al., 2017).

**Table 1:** Main chemical component contents of CFT‐1 and Fuyun6 green tea infusion (mg/ml)

Components (mg/g)	CFT‐1	FYT	Components (mg/g)	CFT‐1	FYT
C	0.27 ± 0.01	0.09 ± 0.01	GC	0.70 ± 0.03	0.35 ± 0.01
EGCG	8.38 ± 0.27	1.10 ± 0.01	Caffeine	2.19 ± 0.02	1.05 ± 0.00
GCG	0.38 ± 0.01	0.03 ± 0.01	Total catechins	17.22 ± 0.35	3.19 ± 0.04
EGC	2.82 ± 0.10	1.27 ± 0.06	Polyphenol	21.57 ± 1.23	6.80 ± 0.95
EC	1.44 ± 0.03	0.32 ± 0.05	Theanine	1.39 ± 0.15	1.46 ± 0.05
ECG	4.31 ± 0.03	0.41 ± 0.01	Polysaccharides	0.12 ± 0.04	0.13 ± 0.02
CG	0.29 ± 0.00	0.04 ± 0.01	Water extract	30.56 ± 1.30	18.47 ± 0.94

Note

Data are averages of triplicate analyses as mean ± *SD* (*n* = 3). Total catechins represent the sum of epigallocatechin (EGC), catechin (C), (−)‐epicatechin (EC), (−)‐epigallocatechin‐3‐gallate (EGCG), (−)‐gallocatechin‐3‐gallate (GCG) and (−)‐epicatechin‐3‐gallate (ECG).

### 3.2 General health condition was not changed by long‐term CFT‐1 consumption

**Figure 2:**
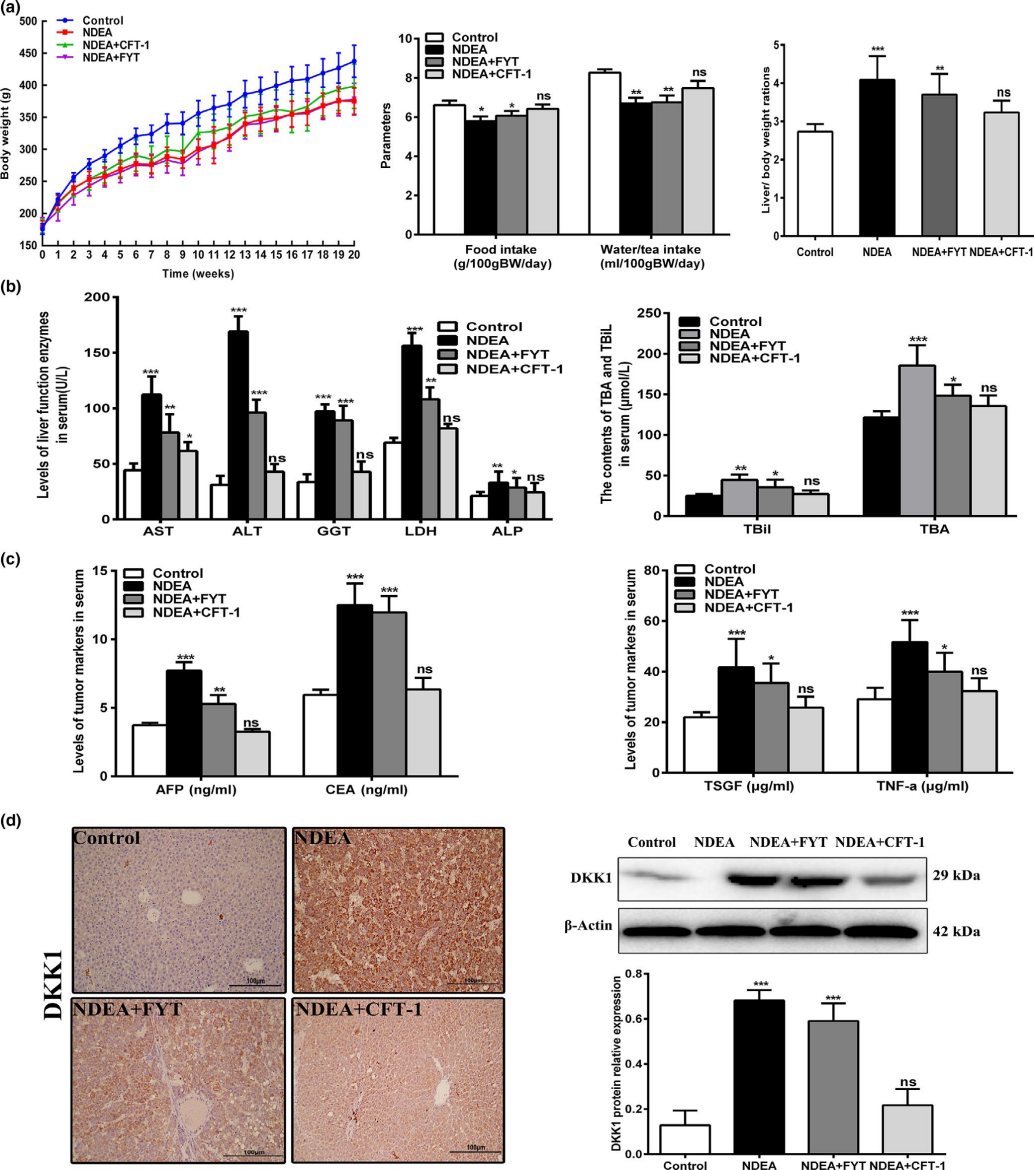
Effects of CFT‐1 and FYT green tea infusion on body weight, fodder intake, water or tea intake, liver/body weight ratios, liver marker enzymes, and serum tumor markers of NDEA‐induced hepatocarcinogenesis rats. (a) The weekly body weights, fodder intake, water or tea intake of each group rats during the experiment and the liver/body weight ratios at 20th week. (b) The liver marker enzymes in serum of control and experimental rats. (c) The levels of serum tumor markers AFP, CEA, TSGF, and TNF‐α in each group rats. (d) The nuclear localization (magnification, ×200) and expression of DKK1 protein in livers of control and experimental rats. Data were expressed as means ± *SD*. *p < 0.05, **p < 0.01, and ***p < 0.001 versus Control. Ns, not significant (*p* < 0.05) versus Control. AFP, alpha fetal protein, ALP, alkaline phosphatase; ALT, alanine aminotransferase; AST, aspartate aminotransferase; CEA, carcino embryonie antigen; DKK1, dickkopf‐related protein‐1; GGT, gamma‐glutamyl transpeptidase; LDH, lactate dehydrogenase; TNF‐α, tumor necrosis factor; TSGF, tumor specific growth factor. Control: normal control group, NDEA: NDEA‐treated group, NDEA + CFT‐1: CFT‐1 green tea infusion plus NDEA‐treated group. NDEA + FYT: FYT green tea infusion plus NDEA‐treated group

DENA administration significantly (*p* < 0.05) decreased water intake, fodder intake, body weight, and increased the liver/body weight ratios in the NDEA group in comparison to control group (Figure 2a). The rats receiving CFT‐1 for 20 weeks did not show significant differences in the fodder and tea consumption with respect to the control group (Figure 2a). Compared with NDEA group, NDEA‐treated group showed markedly (*p* < 0.05) reduction in liver/body weight ratios, while FYT treatment group showed insignificant (*p* > 0.05) change in tea and fodder intake as compared to NDEA group (Figure 2a).

The effects of experimental treatments on blood hematology were summarized in Table S3 of Appendix S1. Hematocrit, leukocyte count, mean corpuscular volume (MCV), platelets, and mean corpuscular hemoglobin (MCH) of NDEA group were significantly higher (*p* < 0.05) than that of control group (Table S3 in Appendix S1). CFT‐1 treatment significantly (*p* < 0.05) reduced WBCs, hematocrit, platelets, MCH, and MCHC but not hemoglobin compared to NDEA group rats and no other organ abnormalities were noted (Table S4 in Appendix S1), suggesting 3% CFT‐1 green tea infusion was nontoxic for rats.

### 3.3 CFT‐1 interventions ameliorated liver damage and decreased tumor markers

Single intraperitoneal administration of NDEA significantly (*p < *0.05) elevated liver function enzymes ALT, AST, GGT, LDH, ALP, TBA, TBil, and levels of tumor markers CEA, AFP, TSGF, TNF‐α in serum compared to normal control rats (Figure 2b,c), suggesting the presence of metabolic disorders in NDEA group animals. CFT‐1 significantly (*p < *0.05) decreased these liver function enzymes and tumor markers, except for AST, indicating a protective effect (Figure 2b). Although all rats exhibited decreased serum liver function enzymes by 20th week, FYT treatment still failed to attenuate the increased GGT and ALT activities depicted by insignificant (*p > *0.05) change compared to NDEA group rats (Figure 2b).

The combined detection of serum AFP and secreted protein DKK1 could be used as an important way to improve the sensitivity and accuracy of clinical diagnosis of primary hepatic carcinoma (Mao et al., 2017; Shen et al., 2015). To determine whether the protein levels of DKK1 changed after treatment with CFT‐1 and NDEA, we examined DKK1 protein expression and its localization in livers by Western blot analysis and immunohistochemical analysis. As shown in Figure 2d, immunostaining to quantify and localize nuclear DKK1 revealed overexpression of DKK1 in liver sections of NDEA group rats, and the protein level of DKK1 was also elevated in NDEA group. However, DKK1 protein expression was significantly attenuated in liver tissues of NDEA + CFT‐1 rats. These results show that the CFT‐1 exhibited inhibiting the production of serum tumor markers DKK1 in NDEA‐induced liver cancer rats.

### 3.4 CFT‐1 exerted a powerful prevention of liver tumorigenesis

**Figure 3:**
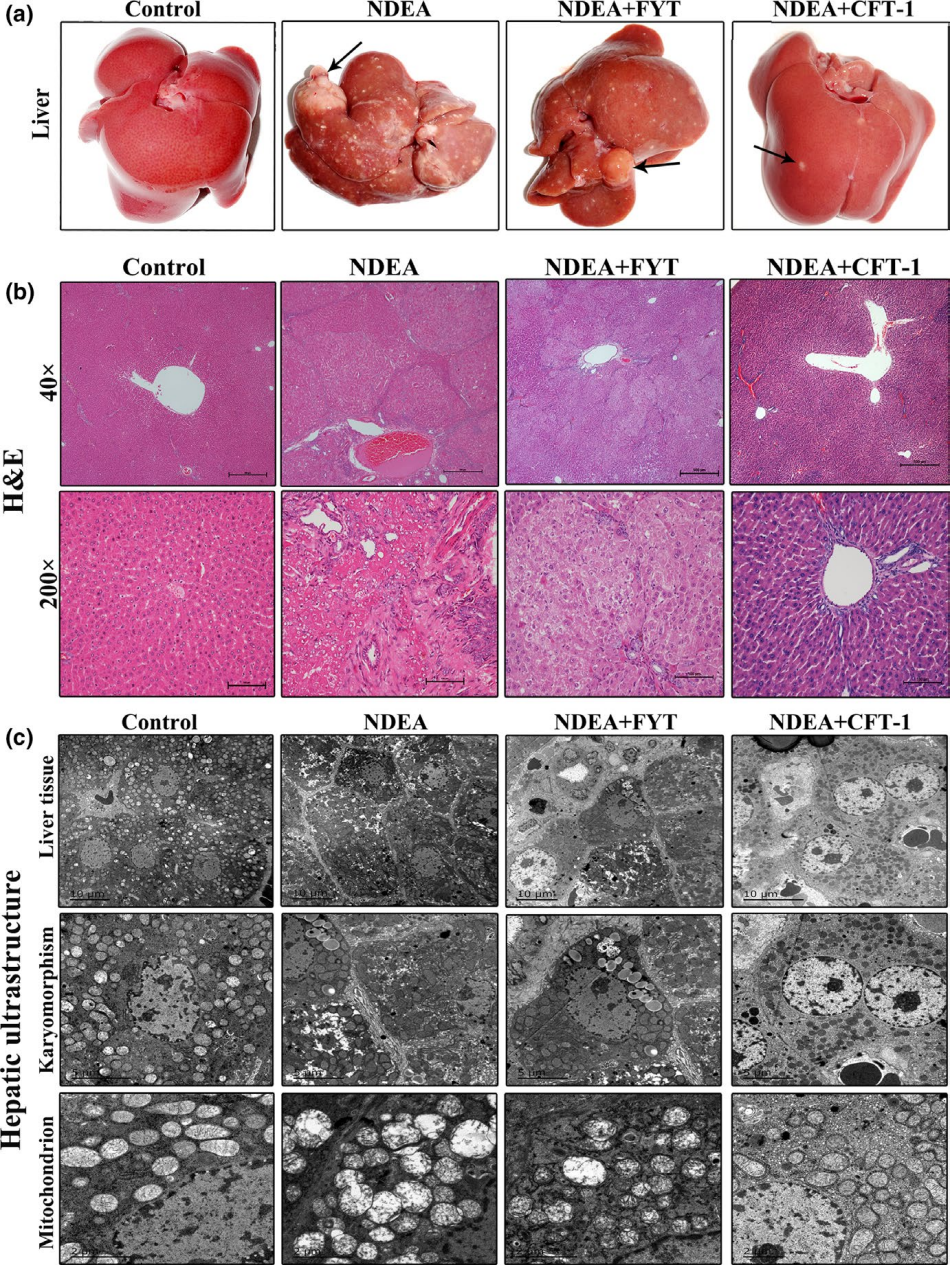
Liver tissue, H&E staining, and hepatocyte ultrastructure of livers in different groups. (a) Representative macroscopic features of the liver tissue in different groups at 20th week. The arrows indicate hepatic nodules. (b) Representative H&E staining images from each group are shown as 40× and 200× magnifications. (c) Hepatocyte ultrastructure features representative transmission electron microscope photomicrographs (Scale bar = 10 μm) and higher magnification images representative hepatocyte karyomorphism (Scale bars = 5 μm) and mitochondria (Scale bars = 2 μm)

Differences with respect to tumors in each treatment group are presented in Table 2 and Figure 3. The control group livers appeared normal in size with smooth surface, whereas severe enlargement of liver with multiple gray‐white nodules was observed in NDEA group (Figure 3a). As shown in Table 2, CFT‐1 significantly reduced the hepatic nodules incidence, size, and number. For example, the max nodule diameter of NDEA group was 28.64 ± 14.31 mm in size, but the nodule size in the NDEA + CFT‐1 group was least as compared to NDEA group (Table 2), suggesting that CFT‐1 could inhibit the development of hepatic neoplastic nodules.

**Table 2:** Effects of CFT‐1 and FYT on hepatic neoplasm‐related lesions induced by NDEA in rats

Hepatic neoplasm‐related lesions	Control (*n* = 12)	NDEA (*n* = 8)	NDEA + FYT (*n* = 10)	NDEA + CFT‐1 (*n* = 12)
Macroscopic lesions				
Nodule incidence^a^ (%)	0/12 (0)	8/8 (100.0)**	9/10 (90.00)	4/12 (33.33)^†^
Max nodule diameter (mm)^b^	—	28.64 ± 14.31**	14.38 ± 5.02^‡^	1.23 ± 1.14^‡^
Average nodule number^a^ (*n*)	ND	66.20 ± 25.65**	26.83 ± 19.52^†^	7.70 ± 6.24^‡^
Microscopic lesions				
HA incidence (%)	0/12 (0)	8/8 (100.0)**	5/10 (50.0)^†^	2/12 (16.67)^‡^
HCC incidence (%)	0/12 (0)	7/8 (87.50)**	4/10 (40.00)^†^	1/12 (8.33)^‡^
Total tumor incidence (%)	0/12 (0)	8 /8 (100.0)**	9/10 (90.0)	3/12 (25.00)^†^

Note

Data are means ± SDs or *n* (%).

Abbreviations: HA, hepatic adenoma; HCC, hepatocellular carcinoma; ND, not detectable.

a Visible nodules (diameter ≥1 mm).

b Max nodule diameter, mean maximum nodule diameter.

Comparisons: compared with control group,

** *p* < 0.01; compared with NDEA group,

^†^ *p* < 0.05;

^‡^ *p* < 0.01 (Fisher's exact test).

Histopathology for normal control rats appeared normal, and NDEA‐treated rats had signs of HCC such as hyperplasia and extensive centrilobular hemorrhagic necrosis, fibrosis, irregular and/or false nuclei, dual‐core cells, apoptosis, mitotic bodies, hepatocyte ballooning, lobular inflammation, and infiltration of macrophages in NDEA group (Figure 3b). CFT‐1 treatment improved hepatic preneoplastic lesions, but aqueous extracts of FYT treatment only marginally modified liver histopathological features (Figure 3b). At the end of the experiment, CFT‐1‐treated animals had the least incidence of HCC (8.33%) followed by FYT (40.00%) and model control rats (87.50%).

The ultrastructure of hepatic cell was examined by transmission electron microscopy. Ultrastructural observation revealed the significant subcellular damage observed in the liver of NDEA group rats (Figure 3c). Specifically, hepatocytes were atypical and cancerous cells were irregular with false or large nuclei dual‐cores, nuclear pyknosis, and hepatic nucleoli volumes were increased, and some hepatic mitochondria proliferated and swelled, dissolved completely, or were altered to vacuolar structures. This was followed by an increase in the amounts of autophagosomes (Figure 3c). FYT treatment caused changes similar to those seen in NDEA group rats, whereas hepatic lesion was ameliorated by CFT‐1 treatment. There were little swelling and degeneration mitochondria in liver tissues of CFT‐1 treatment rats, and the average mitochondrial length (a typical indicator of mitochondrial fission) of CFT‐1 treatment rats was longer than that of NDEA group rats(Figure 3c).

Taken together, these results suggested that CFT‐1 may prevent the early stage of liver cancer and inhibit the development of existing liver cancer in rats.

### 3.5 CFT‐1 enhanced the antioxidant capacity and reduced NDEA‐induced oxidative stress

**Figure 4:**
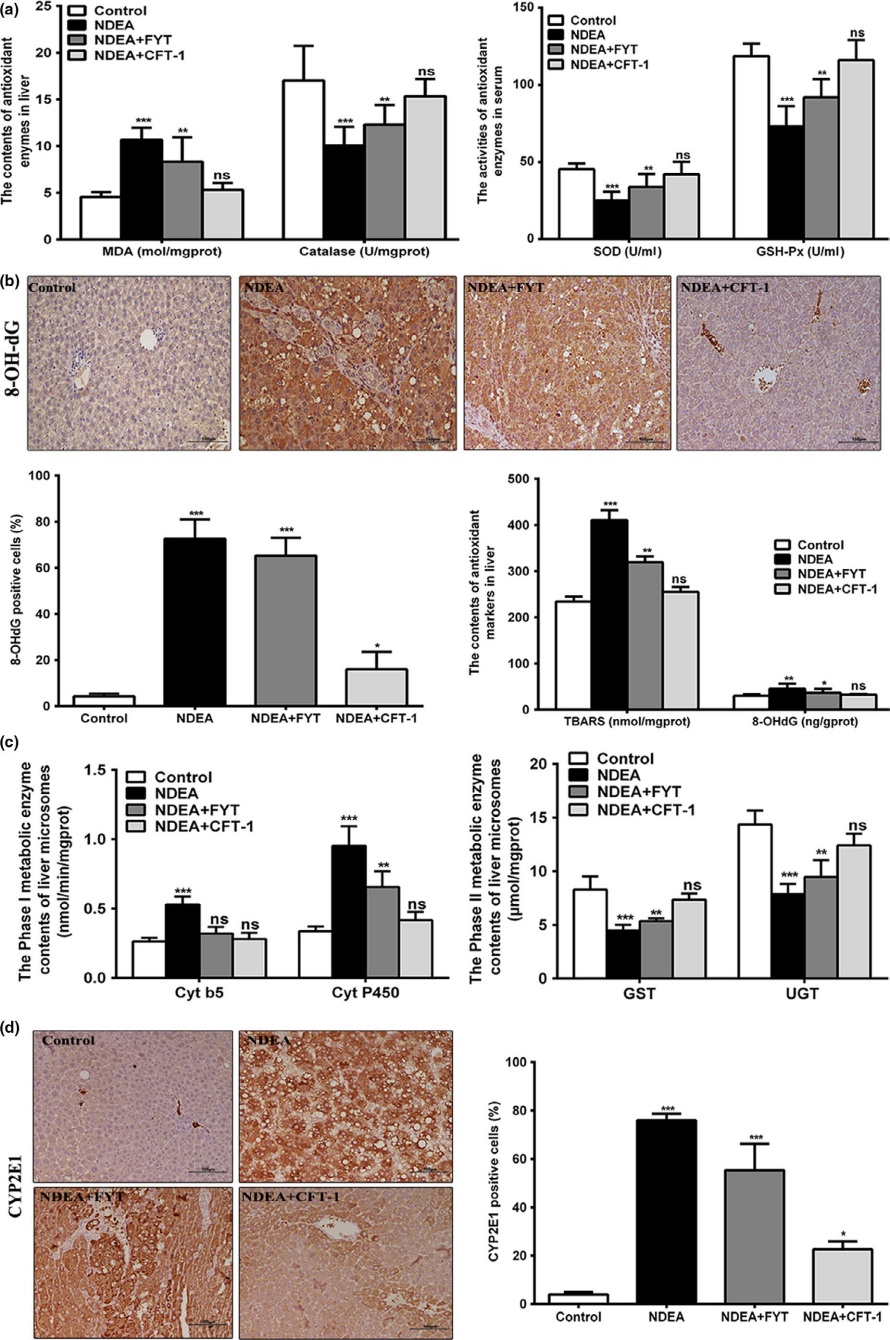
Effects of CFT‐1 and FYT on antioxidant capacity, oxidative damage, and hepatic phase I and phase II detoxification enzyme in NDEA‐induced hepatocarcinogenesis rats. (a) The related antioxidant enzymes activity in serum or liver. (b) The TBARS, 8‐OH‐dG concentrations in livers,and the representative location of 8‐OH‐dG‐positive cells in liver tissues of control and experimental rats. The percentages of positive cells were calculated per 100 cells in 10 HPFs (magnification, 200×). (c) The activity of hepatic phase I and phase II detoxification enzyme in control and experimental rats. (d) The representative location of CYP2E1‐positive cells in liver tissues of each group rats. The percentages of positive cells were calculated per 100 cells in 10 HPFs (magnification, 200×). Data were expressed means ± *SD*. *p < 0.05, **p < 0.01, and ***p < 0.001 versus Control. Ns, not significant (*p* < 0.05) versus Control. 8‐OH‐dG, 8‐hydroxy‐2′‐deoxyguanosine; Cyt b5, cytochrome b5; Cyt P450, cytochrome P450; GSH‐Px, glutathione peroxidase; GST, glutathione S‐transferase; MDA, malonaldehyde; SOD, superoxide dismutase; TBARS, thiobarbituric acid‐reactive substances; UGT, UDP‐glucuronosyltransferase

Fabian et al. (2016) reported that SOD, GSH‐Px, and catalase play important roles in preventing oxidative damage. We measured the SOD, GSH‐Px and catalase activity, and MDA content in serum or liver. As shown in Figure 4a, results demonstrated that serum SOD, GSH‐Px, and hepatic catalase activity in NDEA group was markedly decreased (*p* < 0.001) as compared to control group, while MDA content in livers was markedly increased (*p* < 0.001). However, administration of CFT‐1, serum SOD and GSH‐Px and hepatic catalase activity were significantly increased (*p* < 0.001), while MDA content in livers was markedly reduced compared with the NDEA group (*p* < 0.001). It suggested that CFT‐1 exhibited potent antioxidant capacity against NDEA‐induced hepatocarcinogenesis in rats.

Generally, NDEA treatment may increase the intracellular ROS levels, which can lead to oxidative stress damage (Fabian et al., 2016). 8‐OH‐dG, a product of ROS induced by DNA damage, is an ideal biomarker of oxidative DNA damage (Yarborough, Zhang, Hsu, & Santella, 1996). To evaluate the regulatory effect of CFT‐1 on NDEA‐induced DNA oxidative stress damage, we evaluate the oxidative stress markers TBARS concentrations and the level of 8‐OH‐dG in liver of rats. As shown in Figure 4b, results demonstrated that hepatic TBARS concentrations in the NDEA group rats were significantly increased compared to the control group (*p < *0.001). However, treatment with CFT‐1, the TBARS concentrations were markedly decreased (*p < *0.01) as compared to the NDEA group. The hepatic 8‐OH‐dG level in NDEA rats peaked after NDEA exposure (*p < *0.01) compared to normal control rats. CFT‐1 supplementation significantly decreased the levels of hepatic 8‐OH‐dG compared to NDEA rats, and the difference was statistically significant between CFT‐1 and FYT, indicating that CFT‐1 may modulate the formation of 8‐OH‐dG after NDEA exposure. To verify the production of 8‐OH‐dG in liver tissues, we assayed hepatic 8‐OH‐dG by immunohistochemistry. The 8‐OH‐dG positive cells, with brown 8‐OH‐dG signal in cytoplasm, were diffusely distributed. The results showed that were less in liver of normal control rats, while statistically significantly greater (*p* = 0.0024) in malignant liver tissue of NDEA group rats (Figure 4b). However, the number of 8‐OH‐dG positive cells was significantly decreased in NDEA + CFT‐1 but not NDEA + FYT groups compared to NDEA rats, implying that CFT‐1 could protect livers from oxidative stress damage.

### 3.6 CFT‐1 inhibited the hepatic phase I metabolizing enzymes and induced phase II detoxification enzyme

In NDEA group, the phase I metabolizing enzymes Cyt b5 and Cyt P450 contents increased remarkably at the 20th week (Figure 4c, *p* < 0.001). However, the contents of Cyt b5 and Cyt P450 in the livers of NDEA + CFT‐1 group were remarkably lower than that of NDEA group (Figure 4c, *p* < 0.001). When compared between groups, NDEA + CFT‐1 group had more trend of Cyt P450 suppression than NDEA + FTY group (Figure 4c, *p* < 0.01). The result of the immunohistochemistry also showed that NDEA induced the production of CYP2E1 in liver tissues of NDEA group and CFT‐1 repressed these alterations (Figure 4d).

In this study, the hepatic GST and UGT concentrations in the NDEA group was remarkably (*p* < 0.001) reduced as compared to the control group, which is consistent with earlier reports (Liao, Liu, Xu, & Zheng, 2018). Compared with the NDEA group, the hepatic tissue GST and UGT were significantly increased in the NDEA + CFT‐1 group by 63.94% and 57.95%, respectively (Figure 4c, *p* < 0.001). What's more, the hepatic GST and UGT levels of NDEA + CFT‐1 group were obviously higher than that of NDEA + FTY group (*p* < 0.01), and closed to the normal level. These results demonstrated that CFT‐1 can inhibit the hepatic phase I metabolizing enzymes and enhance the phase II detoxification enzymes activity in NDEA‐induced hepatocarcinogenesis rats.

### 3.7 Identification of differentially expressed genes (DEGs)

**Figure 5:**
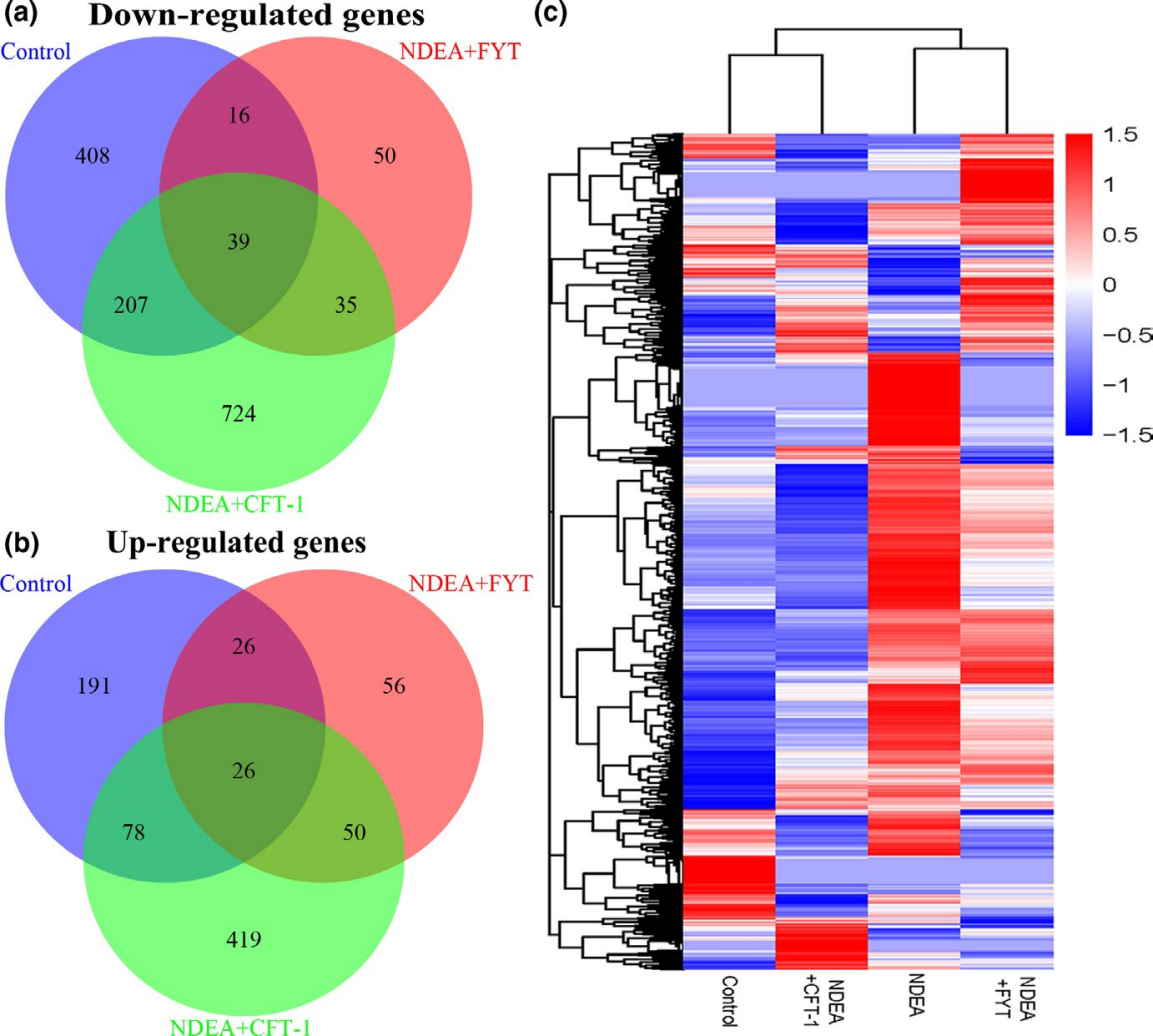
Overview of the genes regulated by CFT‐1 or FYT compared to the model group. (a, b) Venn diagrams displayed the number of down‐regulated genes (a) and up‐regulated genes (b) in livers from the normal control group, NDEA + CFT‐1 group or NDEA + FYT group rats compared to model group. Genes with a *p*‐value <0.05 were counted. (c) Heat map of 6,232 genes with differential expression that appeared in CFT‐1 and FYT treatment groups compared to the model group. The blue represents the gene expression at a low level and red represents the gene expression at a high level

Differentially expressed genes compared to model group are presented in Figure 5, and a total of 34,194 genes changed their expression in at least one sample. Compared to NDEA group, 991 DEGs including 321 up‐regulated and 670 down‐regulated genes were identified in normal liver tissues (Figure 5a,b), suggesting NDEA‐induced liver tumors displayed dramatic gene expression changes compared to normal livers. In addition, a total of 1,578 DEGs were identified by CFT‐1 treatment, including 573 up‐regulated and 1,005 down‐regulated genes, while only 298 DEGs regulated by FYT treatment compared to those of the model group (Figure 5a,b). Furthermore, the heat map color of the CFT‐1 treatment group was overall different than that of the NDEA group and FYT treatment group, and close to that of the control group (Figure 5c). These data indicated that CFT‐1 treatment modulated a larger number of genes or regulated the expression of these genes than treatment with FYT.

### 3.8 Functional classification of DEGs

**Figure 6:**
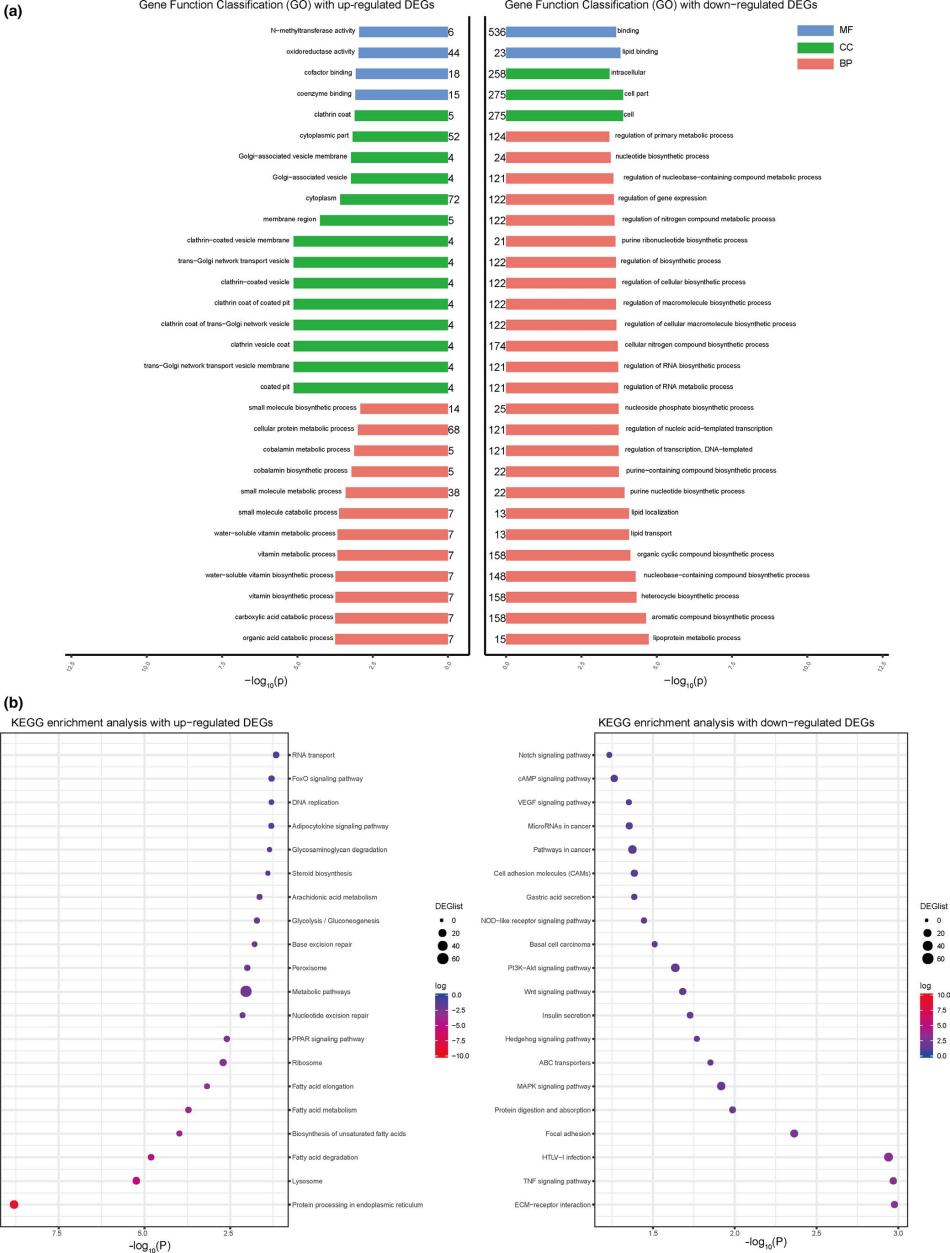
Functional classification of DEGs in NDEA + CFT‐1 group. Gene ontology terms including biological process (BP), cellular component (CC), and molecular function (MF), with *p*‐value <0.05, were regarded as over‐represented categories and were presented in (a). (b) KEGG pathway enrichment analysis was also performed by Fisher's exact test with up‐ and down‐regulated genes in NDEA + CFT‐1 group

To further highlight cellular processes affected by our treatment compared to model group, functional enrichment analyses were performed based on identified DEGs. In NDEA + CFT‐1 group, genes involved in organic cyclic compound biosynthetic process and carboxylic acid catabolic process with oxidoreductase activity and *N*‐methyltransferase activity were significantly up‐regulated, while genes participating into nucleobase‐containing compound biosynthetic process, lipoprotein metabolic process, and lipoprotein metabolic process were down‐regulated (Figure 6a). It is worth noting that many of these GO classes enriched in CFT‐1 treatment group are functionally related to antioxidants, lipid metabolism, lipid transport, lipid localization, ubiquitin/proteasome systems, stress response proteins, xenobiotic‐metabolizing enzymes, kinases and phosphatases, cell adhesion, cell cycle and cell growth, metabolism, movement, transport proteins, and transcription factors, which are closely related to the development of liver cancer. In addition, genes mainly involved in regulation of cell growth and cell communication process were down‐regulated in NDEA + FYT group (Figure S1 in Appendix S1).

The result of canonical pathway analysis in IPA is shown in Figures 6b, S2 and S3. Using a cutoff *p*‐value <0.05, a total of 25 canonical pathways were found to be significantly enriched in NDEA group in comparison with the control group. While compared with the NDEA group, 16 and 13 canonical pathways significantly in livers were enriched after CFT‐1 or FYT‐treated process, respectively (Appendix S2). Interestingly, 9 of the top 20 pathways, including ECM‐receptor interaction, cell adhesion molecules (CAMs), focal adhesion, HTLV‐I infection, MAPK pathway, PI3K‐Akt pathway, protein digestion and absorption, NOD‐like receptor pathway, and TNF signaling pathway, were also recognized in both control group and CFT‐1‐treated group (Figure 6b). These results suggested that these pathways not only play an important role in the NDEA‐induced hepatocarcinogenesis process, but also contain molecular targets that may be regulated by CFT‐1. It is worth noting that when contrasting the NDEA + CFT‐1 compared with the model and the model compared with the control group, some molecules in these pathways showed an opposite direction of change. For example, Tnfrsf1a, Pik3r3, Nod2, Nfkbia, Pik3cd Casp8, and Vegfα in the model versus the control group have significantly up‐regulated, whereas its expression was down‐regulated in livers from the administration of CFT‐1 compared with the NDEA group (Figures S2 and S3 in Appendix S1). These results suggested that CFT‐1 play a preventive role in NDEA‐induced liver cancer though of on the occurrence of may be carried out through these signaling pathways.

### 3.9 qRT‐PCR validation

**Figure 7:**
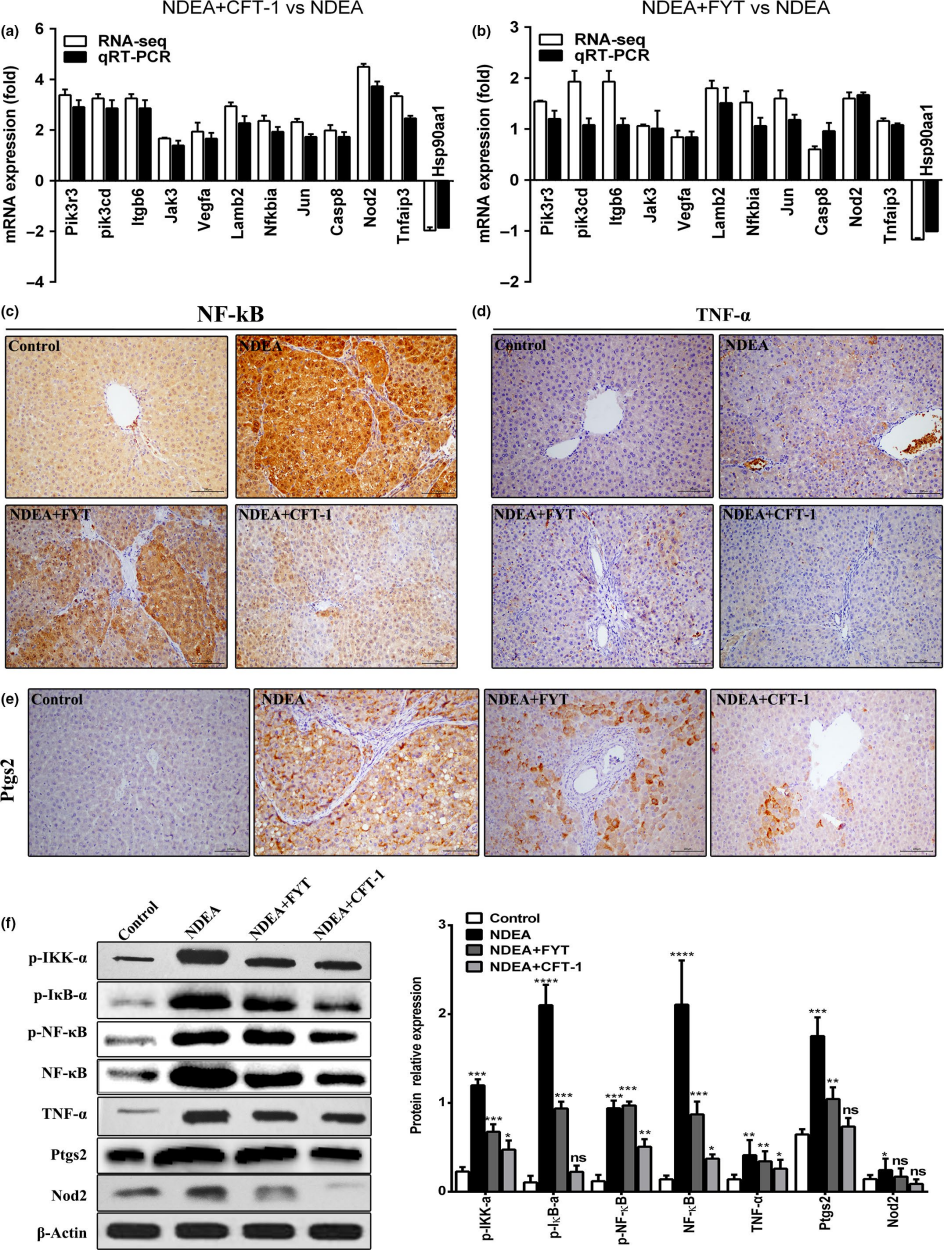
Expression pattern of 12 selected genes as obtained by RNA‐seq and qRT‐PCR, and the effects of CFT‐1 on NF‐κB pathway in NDEA‐induced hepatocarcinogenesis rats. (a) qRT‐PCR validation for the 12 selected regulated DEGs of CFT‐1. (b) qRT‐PCR validation for the 12 selected regulated DEGs of FYT. (c–e) Representative immunohistochemistry for NF‐κB (c), TNF‐α (d), and ptgs2 (e) expression in liver tissue of control and experimental rats (magnification, 200×). (f) Representative Western blot bands and protein relative levels of p‐IKK‐α, p‐IκB‐α, p‐NF‐κB, TNF‐α, ptgs2, and Nod2 in the livers of rats, and their protein relative levels. Data were expressed means ± *SD*. *p < 0.05, **p < 0.01, and ***p < 0.001 versus Control. Ns, not significant (*p* < 0.05) versus Control

To verify the reliability and accuracy of our transcriptome data, we selected 11 up‐regulated and 1 down‐regulated unigenes from the 2 important pathways, phosphoinositide 3‐kinase (PI3K)/Akt and NF‐κB pathway (Figures S2 and S3 in Appendix S1), and evaluated their expression profiles by qRT‐PCR. The expression patterns of selected genes were determined and further compared with those in RNA‐seq assay. Nearly, all of these genes displayed similar expression trend in both techniques (Figure 7a,b), confirming the quantitative properties of the RNA‐seq analysis used in this study.

### 3.10 CFT‐1 inhibited inflammation in NDEA‐induced hepatocarcinogenesis process through NF‐κB signaling pathway

Zheng et al. (2018) have demonstrated that NDEA causes severe chronic inflammatory damage to the liver, thus triggering the development and progression of HCC. Akt can phosphorylate and activate the IκB kinase IKK‐α, causing degradation of IκB and nuclear translocation of NF‐κB where it promotes expression of cytokines, including TNF‐α, accelerating inflammation response (Tang et al., 2016). The immunohistochemistry result showed that a large number of NF‐κB‐positive cells were observed around the central vein and necrotic area in livers of NDEA group rats, and the expression intensity of NF‐κB‐positive cells was significantly higher than those in the control group. NF‐κB‐positive cells were decreased in livers of NDEA + CFT‐1 animals compared to NDEA and NDEA + FYT group (Figure 7c). And also, the treatment of NDEA induced NF‐κB protein expression in livers of NDEA group via Western blot analysis with significantly increased compared with control group (Figure 7f), suggesting activation of NF‐κB and subsequent nuclear translocation. In addition, immunostaining to quantify and localize TNF‐α and ptgs2 revealed limited expression of TNF‐α and ptgs2 in liver sections of normal rats and CFT‐1‐treated rats (Figure 7d,e). In contrast, increased immunoreactivity for TNF‐α and ptgs2 in NDEA group was observed in the liver sections. What is more, the results of Western blot analysis demonstrated that the upstream and downstream signals of NF‐κB, such as p‐IκB‐α, TNF‐α, ptgs2, and nod2 were down‐regulated greatly by CFT‐1 treatment, which were in accordance with the trend of NF‐κB alteration (Figure 7f). These data suggested that CFT‐1 regulated liver cancer progression by inhibiting NF‐κB activation and its downstream signals leading to inflammation response.

### 3.11 CFT‐1 induced apoptosis and inhibition of proliferation through PI3K/Akt pathway

**Figure 8:**
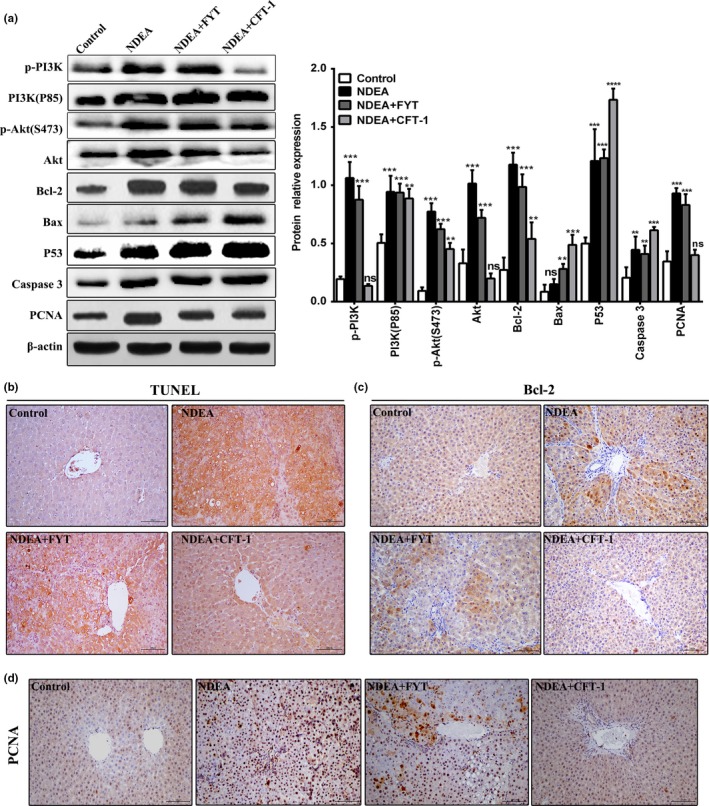
Effects of CFT‐1 on the PI3K/Akt pathway, cell proliferation, and apoptosis in NDEA‐induced hepatocarcinogenesis rats. (a) Western blot analysis for the protein expression of PI3K/Akt pathway (right), and protein levels were normalized to β‐actin (left) in the liver of rats. (b) The results of the TUNEL assay. (c, d) Representative immune‐histochemistry for Bcl‐2 (c) and PCNA (d) protein expression in liver tissue of control and experimental rats (magnification, 200×). Data were expressed means ± *SD*. *p < 0.05, **p < 0.01, and ***p < 0.001 versus Control. Ns, not significant (*p* < 0.05) versus Control

PI3K/Akt pathway is closely related to the occurrence and development of liver cancer, which has become a potential target for the prevention or treatment of liver cancer (Saxena et al., 2007). As shown in Figure 8a, CFT‐1 treatment obviously inhibited the activation of Akt, PI3K and also reduced its phosphorylation. Then, the protein expression of downstream signals, including p53, caspase 3, and Bax protein levels, was elevated, with a decrease of Bcl‐2 protein levels in the livers of NDEA + CFT‐1 group rats as compared with the model group (*p* < 0.01 or *p* < 0.05). Additionally, the results of Western blot analysis showed that the cell proliferation markers PCNA protein expression in NDEA + CFT‐1 group was markedly lower than that of the NDEA group. Finally, we further demonstrate the apoptosis and proliferation responses of aqueous extracts of CFT‐1 interventions during NDEA‐induced hepatocarcinogenesis by the TUNEL method and immunohistochemistry analysis. The results showed that the number of TUNEL‐positive cells was significantly higher in the NDEA group as compared with the CFT‐1‐treated group and control group (Figure 8b). What's more, NDEA rats had observably more Bcl‐2‐positive cells compared with normal control rats (*p* < 0.01). However, CFT‐1 treatment observably (*p* < 0.01) decreased the Bcl‐2‐positive cells (Figure 8c). Additionally, the PCNA‐positive cells were elevated in NDEA group, indicating accelerated proliferation of tumor cells (Figure 8d). However, cell proliferation was significantly inhibited in the CFT‐1‐treated group and suggests an anti‐proliferative effect of CFT‐1. Combining these results, it was concluded that CFT‐1 could activate the PI3K/Akt signaling pathway in occurrence of NDEA‐induced hepatocarcinogenesis. In addition, these results indicate that the apoptosis and cancer cell proliferation processes, which lead to liver cancer in this model, are associated with the PI3K/Akt pathway, leading to the occurrence of apoptosis in liver cancer cell lines.

## 4 DISCUSSION

*N*‐nitrosodiethylamine has been widely used to induce liver cancer in preclinical models. In this study, NDEA‐induced hepatocellular carcinoma model in rats was successfully constructed, and the liver histopathological and ultrastructural observation findings confirmed complete destruction of the hepatocellular architecture and large number of hyperplastic nodules, foci of altered hepatocytes, proliferated and swollen mitochondria, etc. However, CFT‐1 treatment was effective in inhibiting the progression of NDEA‐induced liver cancer, including inhibiting histopathological and ultrastructural changes, reducing the incidence, size, number of hepatic nodules and preventing the hepatic adenoma or HCC formation. In particular, CFT‐1‐treated animals had the least incidence of HCC (8.33%) followed by common tea treatment (40.00%) and model control rats (87.50%). These results indicated that, compared to the common tea, CFT‐1 tea infusion has better inhibitory effect to the occurrence and development of liver cancer. Furthermore, in this study, the blood hematology, serum lipid markers, and organ index analysis indicated that the natural EGCG‐rich green tea infusion and 3% CFT‐1 tea infusion have few toxic side effects on rats when given at 7.62 ml/100 g body weight daily for 20 weeks.

The chemical composition and content of phytochemicals in tea are crucial to its biological activity. As Lou's group (2006) reported, anticancer activity differed by tea varieties given the same content of tea polyphenol in BGC‐803 cells. A mixture of catechins such as polyphenol E were suggested to be more efficient than EGCG alone for inhibiting growth of CNE2, Raji, and SUNE1 cancer cell lines (Ravindranath, Ramasamy, Moon, Ruiz, & Muthugounder, 2009). In this study, the results showed that long‐term consumption of CFT‐1 tea infusion, a natural EGCG‐rich green tea infusion, had better chemopreventive potential against NDEA‐induced hepatocarcinogenesis compared to common tea as indicated by attenuation of the biochemical, morphological, and pathological changes in livers. These cultivar differences may be attributed to the high contents of EGCG or other catechin monomers or synergistic effect among catechins and other polyphenols. In a previous study, we also reported that the inhibition of EGCG‐rich tea on HepG2 cell transplantation tumors in nude mice (68.36%) was more effective than EGCG alone (42.35%) or common tea (18.14%) (Zheng et al., 2016). Therefore, it can be indicated that the anticancer effect of tea leaves may hinge on the types chemical composition, as well as the content and ratio. In view of the foregoing, it may be more practical and more acceptable to the population that increase or decrease the content of certain special functional components in tea and strengthen the health function of tea.

Numerous studies have shown that exposure to NDEA stimulates the production of free radicals through many pathways, which lead to oxidative stress and cell damage (Bilal et al., 2017; Marra et al., 2011). Many types of cancer and its progression have been linked to changes in oxidative stress (Rice‐Evans & Burdon, 1993; Trush & Kensler, 1991). For example, low‐dose NDEA generates free radicals, which induced the formation of liver DNA‐8‐OH‐dG adducts (Nakae et al., 1997) and causes mutations that lead to carcinogenesis. However, excessive free radicals can be eliminated by both enzymatic and nonenzymatic mechanisms in antioxidant defense systems (Fabian et al., 2016). A previous study (Sengottuvelan, Senthilkumar, & Nalini, 2006) indicated that tea polyphenol supplementation enhanced SOD, catalase and glutathione reductase activity in 1, 2‐dimethylhydrazine‐treated rats. In the present study, the serum ALT, AST activity and MDA content, production of liver 8‐OH‐dG were reduced in NDEA‐treated rats after CFT‐1 treatment, while the activity of SOD, GSH‐PX, and catalase was increased, accompanied by inhibiting the phase I enzymes and activating the expression of downstream phase II detoxifying GST and UGT2b1, which then accelerated the elimination of the metabolites of NDEA, thereby achieving the goal of detoxification. Thus, the protective effect of CFT‐1 against liver function damage and anti‐oxidative damage may be due to its high antioxidant activity and detoxification function. These observations are consistent with our findings in rats—CFT‐1 tea infusion treatment animals had less liver morphological and pathological changes compared to NDEA and FYT group rats.

Furthermore, we used the RNA‐seq to study the mechanism of CFT‐1 in the NDEA‐induced hepatocarcinogenesis rats. The results showed that the altered genes were related to the antioxidants, xenobiotic‐metabolizing enzymes, cell cycle, inflammation, cell proliferation, metabolism, etc. and PI3K/Akt, NF‐κB, MAPK and TNF pathway were altered, which are closely associated with the hepatocarcinogenesis. In addition, the results revealed the dramatic alterations in genes associated with the hepatocarcinogenesis by CFT‐1 or FYT treatment. For example, in this study, compared with the NDEA group, there are a total of 1,578 DEGs in the CFT‐1 group, including 573 up‐regulated and 1,005 down‐regulated genes, while only 298 DEGs containing 158 up‐regulated and 140 down‐regulated genes when treat with FYT (Figure 6). Of the genes increased by CFT‐1, 7% (74/1,005) were also down‐regulated by FYT tea infusion (Figure 6a), whereas 13% (76/573) of the up‐regulated genes in livers of CFT‐1‐treated rats was also decreased in the livers of NDEA + FYT group animals (Figure 6b). These results are consistent with our findings in rats—CFT‐1 tea infusion treatment has better inhibitory effect to the occurrence and development of liver cancer compared to the common tea. The GO and KEGG analysis also showed that some of the molecules in the enrichment of biological process and signal pathway showed an opposite direction of change when compared with NDEA + CFT‐1 group and NDEA group, NDEA group, and control group, respectively. It indicated that these biological process and signal pathway was not only related to the occurrence and development of NDEA‐induced liver cancer in rat, but also contain molecular targets that may be modulated by CFT‐1, and some of these genes played an important role in the regulation of NDEA‐induced hepatocarcinogenesis by CFT‐1.

Studies showed that one of the main causes of NDEA‐induced liver cancer is inducing chronic inflammatory response and abnormal repair after liver injury (Mandal & Bishayee, 2015). In this study, we found that the levels of cytokines TSGF and TNF‐α were remarkably increased after exposed to NDEA in the model group compared with control group. However, the levels of TSGF and TNF‐α were remarkably inhibited by CFT‐1 treatment compared with the NDEA group. Zheng Chen and Lu (2011) showed that EGCG, EC, ECG, EGC, and L‐theanine in tea participate in regulating the proteins in the NF‐κB signaling pathway by virtual computer software to mimic molecular docking, and confirmed that tea extract can down‐regulate the expression of multiple target proteins in the NF‐κB signal pathways with certain orientation and conformation, such as cytochrome reductase P450, oxidosqualene cyclase, and NF‐κB. However, the molecular mechanism of these phytochemicals revealing improving liver cancer in vivo remains understood. Thus, we studied whether CFT‐1 could inhibit the hepatocarcinogenesis through NF‐κB pathway. From our study, rats treated with CFT‐1 had down‐regulated hepatic NF‐κB expression. Subsequently, the upstream or downstream signals of p‐IKK‐α, p‐IκB‐α, TNF‐α, ptgs2, and nod2 expression were decreased in protein levels. It indicated that CFT‐1 plays an important role in inhibiting inflammation via regulating NF‐κB pathway, and this may confer HCC resistance during hepatocarcinogenesis induced by NDEA.

The imbalance between cell proliferation and cell death is considered to be a primary pathogenesis of HCC (Khan & Mukhtar, 2013). PI3K/Akt pathway is an important signaling pathway involved in cell proliferation, apoptosis, metabolism, and other functions (Saxena et al., 2007). Therefore, the anti‐apoptosis and anti‐proliferation effects of CFT‐1 via PI3K/Akt pathway were also investigated to further reveal the specific role on anticancer. We observed accelerated apoptosis and decreased PCNA expression after the CFT‐1 treatment in liver cancer cells via immunohistochemistry and TUNEL assay, which is consistent with former studies (Zheng et al., 2016). What is more, Western blot results showed that CFT‐1 treatment obviously inhibited the activation of Akt, PI3K and also reduced its phosphorylation. Then, the expression of p53, caspase 3, Bax, and other regulatory proteins that mainly regulate the apoptosis of liver cancer cells was accelerated, and the Bcl‐2 was down‐regulated after the use of CFT‐1. Further, it was notable that CFT‐1 can inhibit the occurrence and development of liver cancer relying on apoptosis induction and inhibition of cell proliferation by blocking the PI3K‐Akt pathway.

In summary, compared with the common tea, EGCG‐rich CFT‐1 green tea had a superior inhibitory effect in liver carcinogenesis process, especially marked decreased the incidence of hepatic nodules and adenoma or HCC formation induced by NDEA. CFT‐1 can significantly ameliorated abnormal liver function enzymes, improved antioxidant capacity to repair the oxidative damage, regulated hepatic xenobiotic‐metabolizing enzymes, and thus could be a potential ingredient of interests in functional foods. Transcriptomic analysis of liver tissue suggested that administration of CFT‐1 regulated larger genes than the common tea treatment, which were situated in several canonical pathways important in the inflammatory network, antioxidants, xenobiotic‐metabolizing enzymes, apoptosis, cell proliferation, and metabolism related to liver tumorigenesis. Further, it was notable that CFT‐1 inhibited inflammation via inhibiting NF‐κB activation, and inhibited proliferation, and accelerated apoptosis by PI3K/Akt pathway in liver carcinogenesis process.

## CONFLICT OF INTEREST

The authors declare that there is no conflict of interest regarding the publication.

## ETHICAL APPROVAL

The research described herein was performed on male Wistar rats (*Rattus norvegicus*). This study was performed in strict accordance with protocols approved by the Animal Ethics Committee of the Institute of Laboratory Animal Science, Chinese Academy of Medical Sciences, Beijing, China.
