# Supplementary material for: Chemoprevention of elite tea variety CFT‐1 rich in EGCG against chemically induced liver cancer in rats
Source: Food Sci Nutr. 2019 Jul 4;7(8):2647–65. doi: 10.1002/fsn3.1121 (PMC6694417; doi:10.1002/fsn3.1121)
Supplement: Supplementary file 1 [file FSN3-7-2647-s002.docx]

**Appendix A. Supporting Information**

**Chemoprevention of Elite Tea Variety CFT-1 Rich in EGCG against Chemically Induced Liver Cancer in Rats**

Table S1．Sequences of primers used for qRT-PCR analysis.

| UniProt ID | Target gene | Forward primer (5'-3') | Reverse primer (5'-3') |
| --- | --- | --- | --- |
| XM_006238714.3 | Pik3r3 | GCACCTCGTATGGCTCAATC | CACAGAACAAGCGTAACATCCT |
| NM_001108978 | pik3cd | CTTTCTGGGTTGGGTATG | CCACATCAGTGAGAGGAA |
| NM_001004263 | Itgb6 | TGGCAGGCATTGTGATTCC | TCAGACCGCAGTTCTTCGTA |
| NM_012855.2 | Jak3 | TCTTGTAGCCTCTCATCCTCAG | AAGCGTAGCCTGTAGACCAA |
| NM_001110333.2 | Vegfa | TTACTGCTGTACCTCCACCAT | AGGACGGCTTGAAGATATACTCTAT |
| XM_006243709.2 | Lamb2 | CTGTGCTAGGTCTGGTCTCA | TTACACTGACAACGCTGACATC |
| NM_001105720.2 | Nfkbia | TGGTCTCGCTCCTGTTGAA | GCTCTCCTCATCCTCACTCTC |
| NM_021835.3 | Jun | GCCAACCTCAGCAACTTCA | TCTGCGGCTCTTCCTTCA |
| NM_001228.4 | Casp8 | GCAGAGGGAACCTGGTACAT | TCATCCTTGTTGCTTACTTCATAG |
| XM_017601207.1 | Nod2 | GCCTTCCTTCTACAGT | TGGCAGGGCTCTTCTGCAAG |
| XM_017589829.1 | Tnfaip3 | CGGACTCCAGCAGACAGA | CAGAGGCGGTGACAGACT |
| NM_017008.4 | GAPDH | CGGAGTCAACGGATTTGGTCGTAT | AGCCTTCTCCATGGTGGTGAAGAC |

Table S2 Characterization of chemical constituents of CFT-1 and Fuyun6 green tea by UHPLC-Q Extractive Orbitrap-MS.

| Compounds | tR | Identification | Molecular Formula | [M-H]^-^ | MS/MS(m/z) |
| --- | --- | --- | --- | --- | --- |
| 1 | 5.14 | (-)-Gallocatechin (GC)* | C_15_H_14_O_7_ | 305.56^①^(0.25) | 221.01 219.10 125.3 |
| 2 | 7.54 | (-)-Epigallocatechin (EGC)* | C_15_H_14_O_7_ | 305.56(0.27) | 221.18 219.07 179.03 |
| 3 | 9.49 | (+)-catechin (C)* | C_15_H_14_O_6_ | 289.06(0.06) | 203.69 159.12 123.20 |
| 4 | 10.73 | (-)-Epicatechin (EC) * | C_15_H_14_O_6_ | 289.06(0.06) | 203.30 159.65 123.71 |
| 5 | 15.16 | Caffeine (CAF) * | C_8_H_10_N_4_O_2_ | 195.08(0.18) | 195.08 138.20 110.20 |
| 6 | 17.18 | (-)-Epigallocatechin-3-gallate (EGCG)* | C_22_H_18_O_11_ | 457.06(0.08) | 331.07 305.12 169.05 |
| 7 | 18.59 | (-)-Gallocatechin gallate (GCG)* | C_22_H_18_O_11_ | 457.06(0.08) | 331.07 305.00 287.4 169.31 |
| 8 | 22.36 | (-)-Epicatechin gallate (ECG)* | C_22_H_18_O_10_ | 441.07(0.05) | 331.11 289.11 169.11 |
| 9 | 22.70 | (-)-Catechin gallate (CG)***** | C_22_H_18_O_10_ | 441.07(0.02) | 331.56 203.06 169.34 |

***** Compared with a reference standard．CFT-1：*Camellia sinensis* L. cv. CFT-1．

Table S3. Effects of CFT-1 and FYT on hematological parameters in NDEA-induced hepatocarcinogenesis rats.

| Parameter | Control | NDEA | NDEA +CFT-1 | NDEA +FYT |
| --- | --- | --- | --- | --- |
| WBC(×10^3^μL^-1^)  Lymphocytes (%)  Monocytes (%)  Granulocytes (%)  RBC(×10^3^μL^-1^)  Hmatocrit(%)  MCV(fL)  Platelets (×10^3^μL^-1^)  MCH(pg)  Hemoglobin(g/L)  MCHC(g/L) | 7.77±2.90b | 10.25±2.09a | 7.36±2.13b | 8.59±1.89b |
|  | 72.80±3.99a | 67.49±9.64c | 71.11±2.75ab | 69.43±4.72bc |
|  | 2.90±0.36c | 3.27±0.50ab | 3.03±0.49bc | 2.89±0.31c |
|  | 29.42±5.57a | 28.20±3.54ab | 29.31±9.60a | 26.20 ±2.01bc |
|  | 8.05 ±1.97a | 9.18±0.31a | 8.10±1.97a | 8.42±1.74a |
|  | 48.70±12.18a | 53.54±1.95b | 51.19±8.70a | 53.94±3.10b |
|  | 59.83±0.80b | 61.10 ±0.69a | 60.08±0.73ab | 57.94±1.13c |
|  | 849.00±247.99b | 1327.71±489.41a | 862.88±283.19b | 890.00±330.16b |
|  | 17.75±0.46a | 15.31±2.43c | 17.34±0.22a | 16.98±0.53a |
|  | 144.33±16.81b | 155.80±15.48a | 147.50±17.96ab | 153.79±10.99a |
|  | 260.74±19.42c | 291.83±8.53a | 265.00 ±40.23bc | 280.83±7.32ab |

WBC: leucocyte; RBC: erythrocyte; MCV: mean corpuscular volume; MCH: mean corpuscular hemoglobin; MCHC: mean corpuscular hemoglobin concentration. Data are represented as mean ±SD. Different lowercase letters represent statistically significant at *p*< 0.05(Duncan’s test).

Table S4. Effects of CFT-1 and FYT on relative weight of organs in NDEA-induced hepatocarcinogenesis rats.

| Parameter | | Control | NDEA | NDEA +CFT-1 | NDEA +FYT |
| --- | --- | --- | --- | --- | --- |
| Relative weight of organs (g/100g BW) | liver | 2.87±0.17d | 4.00±0.27a | 3.22±1.09bcd | 3.70±0.21ab |
|  | lungs | 0.38±0.06a | 0.34±0.04a | 0.36±0.07a | 0.35±0.03a |
|  | spleen | 0.15±0.01bc | 0.19±0.05a | 0.15±0.01bc | 0.16±0.02bc |
|  | thymus | 0.14±0.07bc | 0.20±0.06a | 0.15±0.02bc | 0.18±0.03ab |
|  | kidney | 0.53±0.03c | 0.71±0.04a | 0.63±0.04b | 0.57±0.18bc |
|  | Adipose | 0.14±0.07bc | 0.20±0.06a | 0.13±0.02c | 0.18±0.03ab |

Data are represented as mean ±SD. Different lowercase letters represent statistically significant at p< 0.05(Duncan’s test).


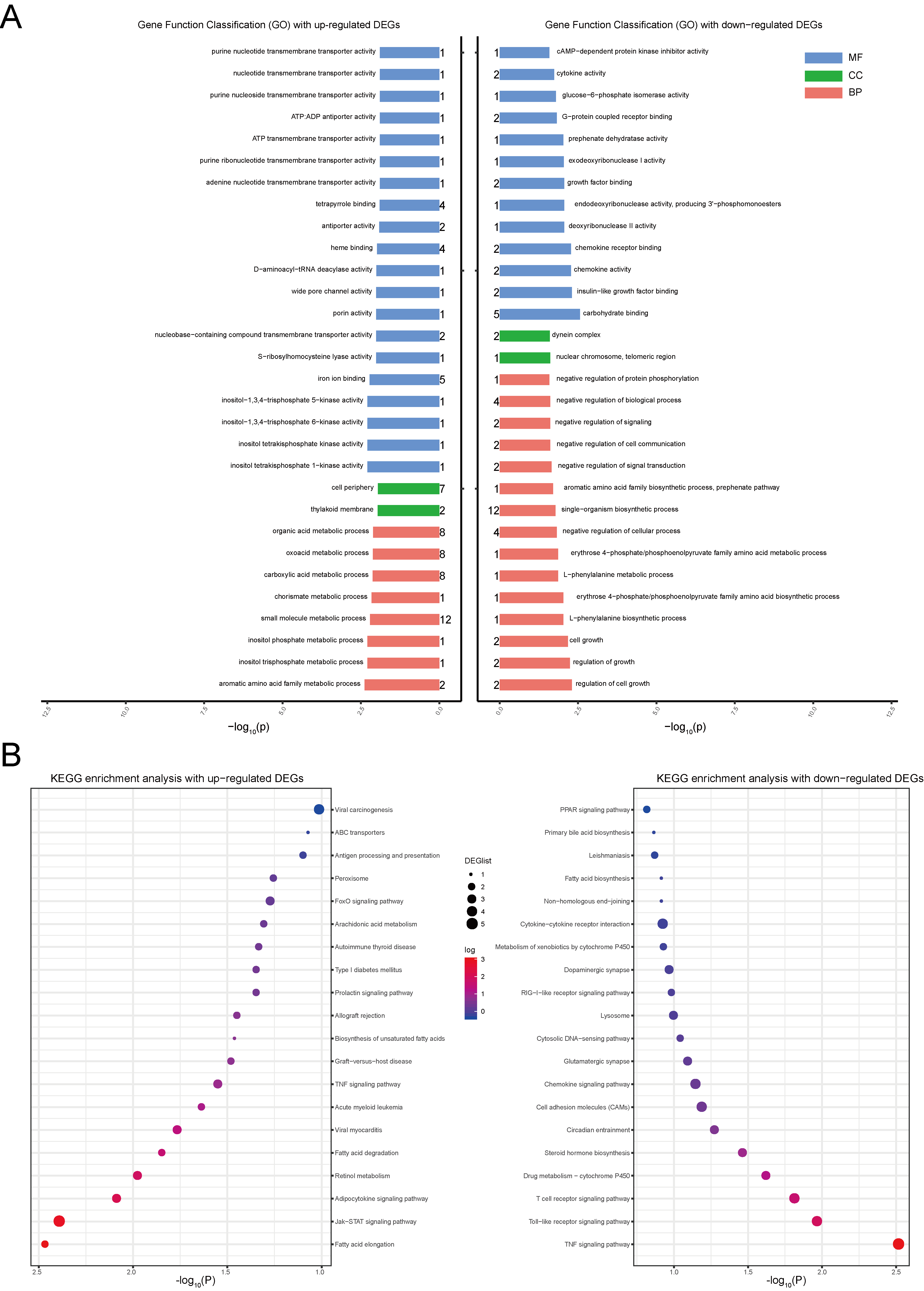


Figure S1 **Functional classification of DEGs in NDEA+FYT group.** Gene ontology terms including biological process (BP) , cellular component (CC) , and molecular function (MF), with p-value < 0.05 was regarded as over-represented categories and were presented in A. B. KEGG pathway enrichment analysis was also performed by Fisher exact test with up- and down- regulated genes in NDEA+FYT group.


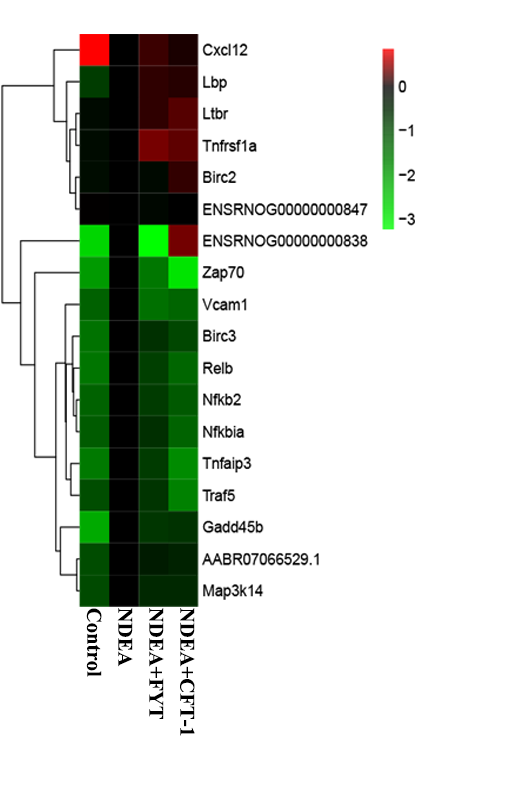


Figure S2 heatmap for log2(fd) compared to model NF-κB signaling pathway


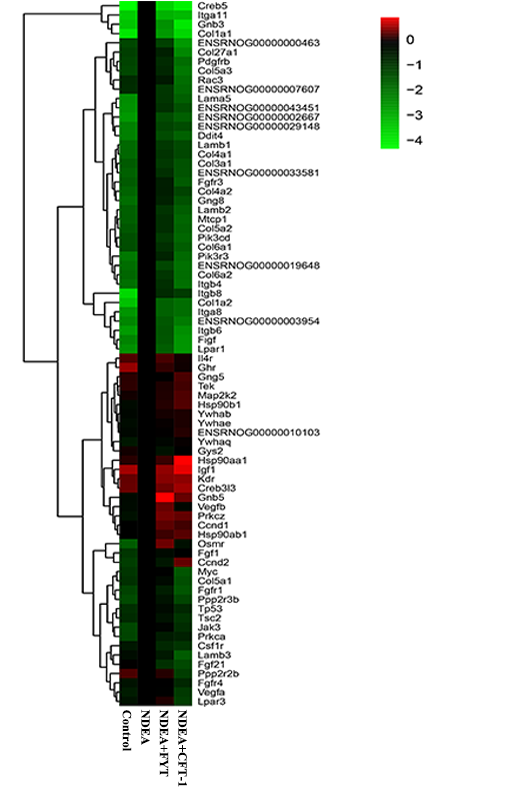


Figure S3 heatmap for log2(fd) compared to model PI3K-Akt signaling pathway ID
